# Shallow endoscopic submucosal dissection using a gas-free immersion system to prevent strictures after esophageal dissections

**DOI:** 10.1055/a-2604-8143

**Published:** 2025-06-18

**Authors:** Tatsuma Nomura, Hiroaki Kumazawa, Takanobu Mitani, Yoshiaki Isono, Tomohiro Sase, Tomonori Saito, Katsumi Mukai

**Affiliations:** 1 Department of Gastroenterology, Suzuka General Hospital, Suzuka, Mie, Japan; 2 Department of Endoscopy Center, Suzuka General Hospital, Suzuka, Mie, Japan

Prevention of strictures after extensive esophageal endoscopic mucosal dissection (ESD) is important 1 2 . Currently, local injection or oral administration of steroids is widely used to prevent strictures; however, there is still no established method. In early esophageal cancer, there is a lymph node metastasis risk if even a small amount of extension is observed in the submucosa; therefore, there is no need for extensive submucosal resection for curable esophageal cancer. By leaving a thick submucosal layer above the muscle layer, it is possible to prevent esophageal stricture caused by damage to the muscle layer due to knife discharge during dissection. Therefore, we propose a submucosal dissection method that intentionally leaves a thick submucosal layer of the esophagus directly below the intrinsic esophageal gland. In this method, we used a gas-free immersion system (GFI). This saline-immersion method uses a 4 mm tapered tip hood to provide a 1.3-fold magnifying effect for precise ESD 3 4 .

The patient was a man in his 60s with a wide early esophageal cancer of the cervical esophagus ( Fig. 1 , Video 1 ). First, we made a circumferential incision, fixed the clip with a line to the normal mucosa on the oral side and applied traction. A tapered-tip CAST hood was then used to accurately dissect the submucosa directly below the muscularis mucosa through saline immersion 5 . Depending on whether the esophageal gland was identified or not, the dissection was performed directly below it or in the shallow submucosa directly below the muscularis mucosa, respectively. The tumor was completely resected within approximately 29 min. Triamcinolone 80 mg was injected locally, and the patient received oral steroids for 6 weeks. The tumor was diagnosed as a squamous cell carcinoma, pT1a. Follow-up endoscopy showed that the cervical esophagus had not narrowed, and the normal lumen width was maintained.

**Fig. 1:**
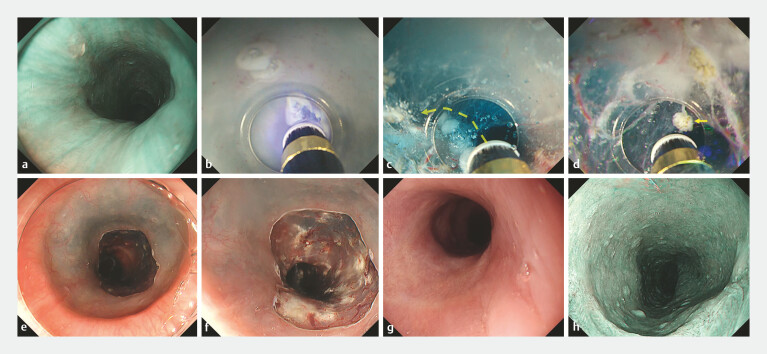
Partial-circumferential ESD of the cervical esophagus using a gas-free immersion system for shallow submucosal dissection. **a** Early esophageal cancer in the cervical esophagus. **b** Incision using a tapered tip hood on the mucosa, which narrowed after the local injection. **c** The submucosal layer shows the intrinsic esophageal gland (yellow arrow). The muscularis mucosa and intrinsic esophageal gland were visualized underwater using an endoscope, and the submucosal layer just below them was dissected. **d** The submucosal layer shows the esophageal gland, and the submucosal layer just below it is precisely dissected (yellow dotted arrow). The submucosal layer remains thick above the muscle layer. **e** Mucosal defect of 50 mm in diameter after ESD. **f** Defect after ESD after local steroid injection. Histopathology revealed that the lesion had been completely resected and that it was a squamous cell carcinoma with a depth of pT1a-LPM. **g, h** Ten weeks later, the ulcer had completely healed, and there was no stricture on endoscopy. Abbreviation: ESD, endoscopic submucosal dissection; LPM, lamina propria mucosa.

Shallow dissection using a gas-free immersion system for submucosal resection of the cervical esophagus and mucosal defects after healing.Video 1

Endoscopy_UCTN_Code_TTT_1AO_2AG_3AD

## Notes

Endoscopy E-Videos https://eref.thieme.de/e-videos
*E-Videos* is an open access online section of the journal *Endoscopy* , reporting on interesting cases and new techniques in gastroenterological endoscopy. All papers include a high-quality video and are published with a Creative Commons CC-BY license. Endoscopy E-Videos qualify for HINARI discounts and waivers and eligibility is automatically checked during the submission process. We grant 100% waivers to articles whose corresponding authors are based in Group A countries and 50% waivers to those who are based in Group B countries as classified by Research4Life (see: https://www.research4life.org/access/eligibility/ ). This section has its own submission website at https://mc.manuscriptcentral.com/e-videos .
